# Factors influencing implementation of digital cardiac rehabilitation: A qualitative analysis of health workers perspectives

**DOI:** 10.12688/hrbopenres.13929.1

**Published:** 2024-07-26

**Authors:** Irene Gibson, Claire Kerins, Louise Foley, Lisa Hynes, Molly Byrne, Andrew William Murphy, Caitlin Marie Reardon, John William McEvoy, Oonagh Meade

**Affiliations:** 1 School of Medicine, University of Galway, Galway, County Galway, Ireland; 2 National Institute for Prevention and Cardiovascular Health, Galway, Ireland; 3 Health Promotion Research Centre, University of Galway, Galway, County Galway, Ireland; 4 School of Allied Health, University of Limerick, Limerick, County Limerick, Ireland; 5 Croí, West of Ireland Cardiac and Stroke Foundation, Galway, Ireland; 6 Health Behaviour Change Research Group, University of Galway, Galway, County Galway, Ireland; 7 Health Research Board Primary Care Clinical Trials Network Ireland, University of Galway, Galway, County Galway, Ireland; 8 Veteran Affairs (VA) Center for Clinical Management Research, VA Ann Arbor Healthcare System, Ann Arbor, Michigan, USA; 9 School of Psychology, University of Galway, Galway, County Galway, Ireland

**Keywords:** Cardiovascular disease; secondary prevention; cardiac rehabilitation; digital health; implementation science; Consolidated Framework for Implementation Research

## Abstract

**Background:**

Despite growing evidence for the effectiveness of digital cardiac rehabilitation (CR) uptake of this technology remains low. Understanding the factors that influence implementation of digital CR in clinical practice is a growing area of research. The aim of this nested qualitative study was to explore health worker perspectives on factors influencing implementation of a digital CR programme.

**Methods:**

Using convenience sampling, semi-structured interviews were conducted with health workers, including health care professionals (nurses, dietitians, physiotherapists) and those in administrative and managerial roles who were involved in delivering and referring patients to Croí MySláinte, a 12-week digital CR intervention delivered during the Coronavirus 2019 pandemic. The updated Consolidated Framework for Implementation Research (CFIR) guided data collection and framework analysis.

**Results:**

Interviews were conducted with 14 health workers. Factors influencing implementation of Croí MySláinte were multiple, with some operating independently and others in combination. They related to: (i) characteristics of individuals (e.g., senior leadership support, commitment and motivation of Health workers to meet patient needs, technical capability, workload and perceived fit with role); (ii) features of the programme (e.g., accessibility and convenience for patients, the digital platform, patient self- monitoring tools, the multidisciplinary team and core components); (iii) the external environment (e.g., partnership and connections between organisations, broadband and COVID-19); (iv) the internal environment (e.g., organisational culture, teamwork, resources including funding, digital infrastructure and staffing); and (v) the implementation process (e.g., engaging patients through provision of technical support).

**Conclusion:**

The study findings suggest that factors influencing implementation of digital CR operate at multiple levels. Therefore, multi-level implementation strategies are required if the true potential of digital health in improving equitable cardiac rehabilitation access, participation and patient outcomes is to be realised.

## Introduction

Secondary prevention is an important strategy to reduce the burden of cardiovascular disease (CVD), which accounts for almost one third of all deaths globally ^1, 2^. Comprehensive secondary prevention programmes, which include cardiac rehabilitation (CR), can reduce CVD mortality, recurrent CVD hospital admissions, and improve overall quality of life ^3, 4^. However, CR is underutilised, with barriers including low referral rates, lack of access and limited capacity, among others ^5, 6^. Notably, the majority of eligible patients do not meet guideline recommended lifestyle and risk factor targets ^7, 8^. Digital cardiac rehabilitation (CR) interventions such as telemedicine, mobile health (smart phone apps, wearables, text messaging), web based applications and virtual reality are emerging as a promising solution to improve uptake and participation ^5^. There is growing evidence to show similar efficacy on important endpoints such as LDL cholesterol, exercise capacity, and medication adherence, which are comparable to in-person, centre based rehabilitation ^9– 11^.

Despite the potential benefits of digital CR interventions, their adoption into routine clinical practice has been slow and most do not reach large implementation scale ^12, 13^. Even during the Coronavirus disease-2019 (COVID-19) pandemic, when technology was urgently needed and embraced, less sophisticated technologies such as the telephone were most widely used to deliver remote CR ^14^. Barriers to the implementation of digital CR are complex and exist at the health system, patient and health workforce level ^15^. Health workers play a pivotal role in bridging the gap between innovative solutions and patient care, ultimately influencing whether a new technology succeeds or fails ^16, 17^. Therefore understanding their experiences is an important part of digital CR evaluation ^17^. However, in comparison to patients, health worker related barriers and facilitators to digital CR ^18^, have been less frequently described with one of the first systematic reviews in this field recently being published ^15^. While this review included 20 studies, from 5 countries, the majority of studies were surveys (n=13) with the remainder (n= 7) focusing on implementation of home-based (n=4), hybrid (combined in person and home based or digitally enabled models, n=1) and virtually delivered interventions (n=2) ^15^. Given that the effectiveness of digital interventions are highly dependent on context ^19^, there remains a need to expand the number of implementation studies focusing on health workers perspectives of specific digital CR interventions. Insights into these perspectives are critical for optimising implementation strategies for digital CR, to enable scale up and translation into clinical practice.

Having already examined the clinical outcomes of “MySláinte” a digital CR intervention delivered during the COVID-19 pandemic ^20^, we conducted a nested qualitative study, to understand what factors influenced implementation of this programme from the perspective of health workers. The aims of this qualitative study were to a) conduct a post implementation evaluation of the barriers and facilitators to “MySláinte” and b) to identify potentially salient factors to inform the future implementation of digital CR. We use the term “health workers” to describe the roles of both health care professional (HCPs) and those in administrative and managerial positions.

MySláinte (Gaelic for MyHealth) was developed by Croí, an Irish Heart and Stroke patient organisation in response to the need to provide patients with access to comprehensive CR during the COVID-19 pandemic. The process of developing MySláinte, together with the key intervention features and clinical outcomes have been previously reported ^20^. In brief, the core components included lifestyle modification, risk factor management, and psychosocial and behavioural change support. Patients received access to a bespoke web-based platform, a Fitbit™, a home blood pressure monitor, and were invited to attend weekly, online group-based supervised exercise sessions and educational workshops. 

## Methods

### Study design

A nested qualitative design using semi-structured interviews was used to examine barriers and facilitators to the delivery of a digital CR intervention. The updated Consolidated Framework for Implementation Research (CFIR) was used to guide qualitative data collection and analysis. The CFIR is a comprehensive, meta-theoretical framework that is used to predict or explain the barriers and facilitators to implementation effectiveness, in health systems at multiple levels ^21^. The CFIR contains 48 constructs and 19 sub-constructs across five domains: 1. Innovation, 2. Outer Setting, 3. Inner Setting, 4. Individuals, and 5. Implementation Process. Table 1. outlines how the CFIR was operationalised for this current study ^21^. As one of the most highly cited frameworks in implementation science, the CFIR has been widely used in digital health research ^22^.

**Table 1.** Operationalisation of CFIR.

CFIR Domain	Definition
Innovation	The Croí MySláinte programme. This domain includes factors related to innovation: design and core components; complexity (number of steps involved in delivery); relative advantages to current practice; evidence base; and the credibility of the HCP team at the source of the programme
Outer Setting	The broader extrinsic ecosystem, in which the Inner Setting exists. This domain includes factors related to policies and laws; critical incidents such as COVID-19; local technological conditions such as availability of broadband, and partnerships and connections between the Inner Setting organisations.
Inner Setting	The setting in which Croí MySláinte was implemented, including both Croí, the heart and stroke patient organisation that directly delivered the programme, and the various hospitals who referred patients and conducted pre- and post- programme assessments. This domain includes factors related to structural characteristics (staffing levels); available resources (funding); access to knowledge and information; compatibility with existing workflows; and tension for change.
Individuals	Roles and characteristics of individuals involved in implementing, delivering, and/or receiving the Croí MySláinte programme. This domain includes factors related to the health workers involved in direct and indirect programme delivery, high-level leaders (cardiologists and senior management), IT support, and family members and patients who received the programme. Characteristics relate to individual needs, capability, opportunity, and motivation.
Implementation Process	The activities and strategies used to implement Croí MySláinte. This domain includes factors related to forming a team, planning, engaging health workers and patients, and making programme adaptations.

Ethical approval was obtained from the Galway Clinical Research Ethics Committee (Ref. C.A 2689) on the 27 ^th^ of August 2021. The study is reported in accordance with the Consolidated Criteria for Reporting Qualitative Research (COREQ) (Supplementary file 1: https://osf.io/z9msx/) ^23^.

### Participants

Using convenience sampling, health workers with various roles and responsibilities for implementation and/or delivery of MySláinte were invited to participate in the study via email by the study partner Croí. Participants included health workers at Croí who delivered the programme (direct deliverers) and health workers from hospital cardiology and CR centres across five sites in the West of Ireland who referred their patients and/or conducted pre and post-programme assessments (indirect deliverers). We aimed to recruit a sample of 10–15 health workers. Sample size estimates were informed by the concept of information power ^24^ and by the number of potential participants available to recruit.

### Data collection

Following informed written consent, which involved providing participants with a description of the research along with the participant information sheet, semi-structured interviews were conducted online between October and December 2021. In acknowledging the lead investigator’s (IG) positionality as a CVD nurse involved in the delivery of MySláinte, interviews were conducted by another study team member (LF). This helped to minimise potential power differentials during data collection, which may have occurred due to existing relationships between the lead investigator and health workers ^25^. The interview guide was informed by the CFIR and was refined through input from a Public and Patient Involvement (PPI) panel of HCPs (n=5), all with experience of delivering digital CR programmes (Supplementary file 2: https://osf.io/z9msx/). The interview guide was piloted in advance with members of the study team, and minor refinements were made prior to interview commencement.

### Data analysis

Using NVivo R1 software for data management, framework analysis ^26^ was performed using the CFIR as the *a priori* framework. To determine if modifications to the coding frame were required, open coding was conducted on a small sample of transcripts. Following this, a deductive approach was adopted to code barriers and facilitators using the framework. In addition, participant views on factors to help inform the future implementation of digital CR were captured using thematic analysis ^27^ and were mapped to the CFIR. Two investigators (IG and CK) independently coded two transcripts, checking for coding consistency and modifying CFIR definitions as necessary. The remainder of the data was analysed by the lead investigator (IG) and a third investigator (CR), a member the CFIR development group, provided expert input on CFIR construct definitions as required.

## Results

Of the 15 health workers invited to participate, 14 (93%) responded. Interviews lasted on average 46 minutes (range 32-50 minutes). Participants included Croí HCPs (nurse prescriber, dietitian, physiotherapist) an administrator and chief executive officer (CEO) (n=5), and hospital cardiology and CR nurses (n=9). Characteristics of participants are outlined in Table 2.

**Table 2.** Baseline characteristics of participants.

	Participants (n=14) n (%)
**Gender**, female	12 (86%)
**Professional role**	
Clinical Nurse Specialist	6 (43%)
Clinical Nurse Manager	1 (7%)
Advanced Nurse Prescriber	1 (7%)
Cardiac Rehabilitation Co-ordinator (nurse)	2 (14%)
Physiotherapist	1 (7%)
Dietitian	1 (7%)
Administrator	1 (7%)
Chief Executive Officer	1 (7%)
**Years working in health**	
0-10	3 (21%)
11 to 20	5 (36%)
21 to 30	2 (14%)
30 or more	4 (28%)
**Service type**	
Community Cardiac Rehabilitation (Direct Deliverers)	5 (36%)
Hospital Cardiac Rehabilitation (Indirect Deliverers)	5 (36%)
Hospital Cardiology Department (Indirect Deliverers)	4 (28%)

**Figure 1.**
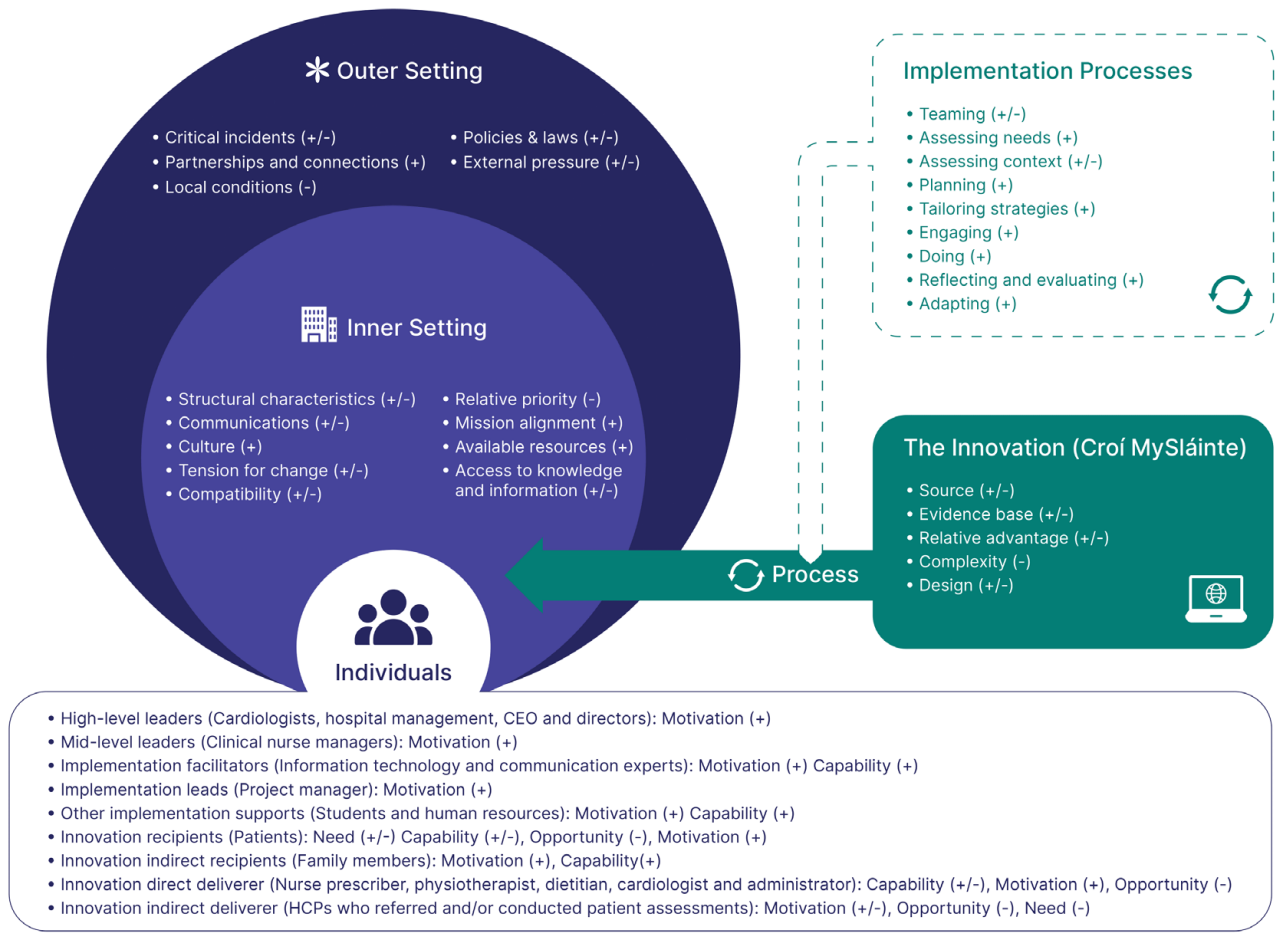
Factors influencing implementation of Croí MySláinte across multiple levels. Note: +, facilitators: -, barriers. Outer Setting = the extrinsic ecosystem, in which the Inner Setting exists; Inner Setting = the setting in which MySláinte was implemented; Individuals = roles and characteristics of those involved in implementing, delivering and/or receiving MySláinte; Innovation = the Croí MySláinte programme; Implementation Process = activities and strategies used to implement MySláinte. This figure has been reproduced with permission from CFIR 2.0. Adapted from Damschroder, L. J., Reardon, C. M., Widerquist, M. A. O., *et al*. (2022). The updated consolidated framework for implementation research based on user feedback. *Implementation Science, 17*, 75. https://doi.org/10.1186/s13012-022-01245-0. Image adapted by The Center for Implementation, © 2022. Version: V2024.01. https://thecenterforimplementation.com/toolbox/cfir

Factors influencing implementation of the MySláinte programme operated across the five CFIR domains: (1) Innovation; (2) Outer Setting; (3) Inner Setting; (4) Individuals; and (5) Implementation Process ( Figure 1). Most factors acted simultaneously as barriers and facilitators, and while many acted independently in influencing implementation, some acted in combination. Through our analysis, we developed a matrix combining health workers perceived barriers and facilitators to implementation. Supported by sample quotes, this matrix also includes considerations to guide the future implementation of digital CR (Supplementary file 3: https://osf.io/z9msx/). The following section provides a narrative summary of the most commonly occurring factors and how they manifested across the five CFIR domains. HCP perspectives on considerations for future implementation efforts of digital CR are incorporated throughout.

### Domain: Innovation

The design of the MySláinte programme acted as both a barrier and facilitator to implementation. Many of the programme features, which facilitated implementation, were compared favourably by Health workers to hospital-based CR programmes. Perceived advantages included increased accessibility and convenience for patients to attend, and while this was identified as particularly important during COVID-19, the need to deliver digital CR programmes beyond the pandemic was emphasised. Furthermore, health workers identified the advantages of having a multidisciplinary team (MDT) including a nurse prescriber and a dietitian, which is not routinely available in many hospital-based CR programmes. Virtual care was described as being more individualised due to the use of self-monitoring tools, patients being in their own environment, and the ability to involve the family. All of these programme features were identified as important in engaging patients to participate and subsequently, achieving positive patient outcomes.

*With virtual it just makes it more easily accessible. Also some people were carers, some didn’t have transport. They were minding family, minding kids, minding partners so you know they could just do it from the comfort of their own home. (P11, Referrer)*

The programme evidence base, together with the professionalism, reputation and credibility of the MySláinte team who developed the programme, were identified as positively influencing implementation. These factors helped to secure programme funding and importantly helped to engage patients to participate and Health workers to refer.

*While we were hesitant about it being online, we were confident in Croí because of their experience and reputation for delivering high quality prevention programmes. (P7, Referrer)*

Poor functionality of the online platform, together with an increased workload for Health workers, acted as barriers to implementation. For example, Health workers reported managing a larger volume of patients, having additional paperwork, and a general lack of compatibility between virtual and existing work practices, which added to the complexity of implementing the programme.

*We had to rethink how you actually did everything because maybe one step in the real world could equal two or three or even four steps in the virtual world.* (P9, Croí HCP)

Furthermore, referring Health workers identified challenges associated with supporting end of programme assessments. Having not been involved in direct programme delivery, these challenges related to a lack of familiarly with the patient, which was exacerbated by limited access to their clinical information.

Relative to hospital-based CR programmes, identified disadvantages included the inability to monitor patients in the absence of telemetry, and the impact of limited face-to-face contact on peer support and the patient relationship.

*To me there’s a huge void you know if there is no telemetry. I do think they need to be on telemetry to identify problems post event, heart blocks and new A-fibs and all of that. (P5, Referrer)*

### Domain: Outer setting

As a critical incident, the COVID-19 pandemic largely facilitated implementation of MySláinte. It created the momentum for change, increasing patient and HCP engagement in programme implementation.

*I think the fact that COVID happened we were pushed to do things that we’d always wanted to do a bit quicker*, it *forced us all online, even patients and made the uptake of these things easier.* (P1, Croí HCP)

However, local conditions, specifically the lack of broadband was identified as a major barrier to implementation and a source of inequity in terms of patient access, with Health workers recommending that:

*Broadband should be a fundamental right and nobody should be left behind in terms of the digital age.* (P9, Croí HCP)

Strong partnerships and connections facilitated implementation, with referring Health workers speaking to the positive relationships that had developed between organisations (referring hospitals and Croí) through previous projects and how this helped to engage them and increase their confidence in referring to MySláinte. Information sharing and co-learning from other CR centres beyond those involved in the study also helped inform implementation efforts.

*Our organisations had a good relationship … we would have met face-to-face on a couple of different initiatives that we did together so we kind of knew each other and that helped. (P8, Referrer*)

### Domain: Inner setting

The Inner Setting where Health workers worked (Croí and the referring hospitals) exerted both a positive and negative influence on implementation. Limited access to hospital-based CR services due to the COVID-19 pandemic and ensuring patient needs were met created the tension for change, which facilitated implementation. Related to this, MySláinte was seen as compatible with the organisations mission, culture and service priorities to address waiting lists for CR.

*As an organisation our role is to respond to patients needs and our raison d’etre for delivering this programme was to make sure that these patients recieved this vitally important care and support. (P14, Croí HCP)*
*It was desperately needed. The waiting lists for rehab, they’re still really bad, but they were very bad back then because there was nothing happening and people were still having events. (P4, Croí HCP)*

Access to available resources such as funding, the digital platform, and knowledge and information to deliver MySláinte facilitated implementation, and were highlighted as critical to future implementation efforts.

*They need to come up with all the resources, I.T., manpower, funding, everything … if you’re establishing a program like this ... that planning has to be there before it is implemented. (P13, Croí HCP)*

For referring Health workers, the existing work infrastructure acted as a barrier to implementation, with low staffing levels decreasing their opportunity to refer patients. Furthermore, referring patients to MySláinte became an additional task and less of a priority with the return of in person programmes.

*We were short staffed as we were being redeployed and referring was time-consuming … especially when we were starting back with our own groups again. (P10, Referrer)*

### Domain: Individuals

Individuals involved with implementing, delivering, and/or receiving MySláinte are outlined in Figure 1. Health workers identified how the commitment and support from many individuals, ranging from high-level leaders (Cardiologists, Hospital Management, Nursing and CEO’s) to implementation project managers, to IT support facilitated implementation.

*Everybody just seemed to row in together you know and get things done that would have taken months previous to COVID, it just happened. (P5, Referrer)*

While Health workers described barriers having to navigate new technology with limited training, they were highly motivated to deliver MySláinte. This was influenced by their knowledge and beliefs regarding the positive consequences for patients, and their service (e.g., reduced waiting lists), and fit with their social/professional role and identity.

*We all had the patient’s best interests and we all wanted to do something and make a difference … when we saw it was working ... their blood pressure improved, their cholesterol improved ... that gave us confidence. (P4, Croí HCP)*

Time constraints impacted negatively on the opportunity to deliver the programme. Health workers reported that the process of using the technology, determining patient suitability (contacting patients, accessing medical information, risk stratification), coupled with increased administrative duties, was time consuming. Access to patient information through more integrated technology information systems and greater administrative support were identified as solutions to overcome these barriers.

*Invest in the technology to ensure online delivery is more streamlined ... we need to be able to share information across organisations … to ensure patient centred, integrated care. (P1, Croí HCP)*

Health workers reported that while the programme responded to unmet patient needs regarding access to CR and peer support, there was a sense of uncertainty regarding who digital CR interventions are suitable for. This uncertainty related to the patients CVD risk profile and safety, their socio-demographics, and the potential to disenfranchise those with low digital literacy levels.

*People mightn’t necessarily have a smart phone, they mightn’t have a laptop and those kind of digital inequalities and financial constraints can be an issue. (P3, Referrer)*

### Domain: Implementation process

The strategies used to implement MySláinte mainly acted as facilitators to delivery. Health workers highlighted how efforts to engage patients, through the provision of information and technical support, increased the patient’s capability and confidence to participate.

*Where people didn’t have the necessary skills, but they had the technology, they actually went and trained them. (P14, Croí HCP)*

Referring Health workers reported that they were effectively engaged through good relationships with Croí. There were multiple meetings, with demonstrations on the digital platform, and they felt their expectations and concerns were acknowledged. This was identified as an important factor to support future implementation.

*If you’re looking to innovate and support you need to try and engage as much as you can in advance and not just land something on people. And be sensitive to all these fears and anxieties that people will have. (P7, Referrer)*

The commitment and motivation of the team, coupled with a project plan where roles and responsibilities were agreed in advance positively influenced the implementation process. Furthermore, through reflecting and evaluating, MySláinte was adapted iteratively in response to patient needs.

*Some of the first education sessions we did we were like ... these are a bit long ... so we tried to make them more interactive … we were constantly changing and tweaking it. (P14, Croí HCP)*

## Discussion

Using the CFIR, this study examined health worker perspectives of factors influencing implementation of MySláinte, a digital CR intervention delivered during the COVID-19 pandemic. Established areas of focus for implementation of digital health interventions (DHIs) include: intervention characteristics and technical factors, individuals (workforce and patients), the healthcare ecosystem and the broader extrinsic ecosystem within which they operate ^19, 28^. Consistent with recent systematic review and scoping review evidence ^13, 15, 29^, we identified barriers and facilitators to implementation across all of these areas, including the implementation process which often receives insufficient attention in digital technology implementation frameworks ^16^. To our knowledge, this is the first study to explore health worker perspectives of digital CR using the CFIR. While most factors influencing implementation of MySláinte operated independently, many also acted in combination. For example, strong partnerships and connections at the outer setting between organisations combined with the programme features (e.g., inclusion of the MDT and recommended CR core-components) and evidence base increased health worker confidence, and subsequent engagement in implementation. Consistent with previous studies ^16, 22, 29^, these findings demonstrate the dynamic inter-relationships that exists between factors. Therefore, understanding these inter-relationships is essential to optimising implementation of digital CR ^22^.

The perceived usefulness of technology has been identified as one of the most common facilitators to implementation of DHIs among health workers ^13, 29^. In keeping with these findings, the perceived value of MySláinte in providing patients with essential CR, whilst also addressing CR service waiting lists, motivated health workers to engage in implementation. Engagement was further enhanced by organisational leadership support and a culture of patient centeredness, both established predictors of implementation success ^21, 22^. These findings reaffirm that implementation of DHIs is a social process which is influenced by the values, mind-set and engagement of individuals involved, including local champions ^16, 30^. Health workers identified multiple benefits associated with MySláinte (e.g., increased accessibility and convenience for patients) which superseded in person CR programmes. However, health workers acknowledged that there was a trade-off between these benefits and perceived programme disadvantages, for example lack of peer support. In line with WHO recommendations, these findings emphasise the need for the “value” of digital CR to be clearly communicated, including benefits for patients, health workers, and health systems as well as the rationale for why they are superior to the current standards of care ^17, 31^.

The implementation of MySláinte required a significant shift in how health workers delivered CR care with new work practices and care pathways needing to be established. Not unique to MySláinte, the rapid transition to remote care during the COVID-19 pandemic necessitated the use of an existing platform Moodle, which was not appraised as optimal for CR delivery ^14, 32^. This led to an increased workload, which added to the complexity of delivering CR care. Increased work and altered workflows are frequently cited barriers among health workers to DHI implementation ^29^, including those specific to CR ^15^. To address these barriers, there is a need to involve health workers in all stages of the design, development and implementation of digital CR ^19, 33, 34^. Indeed, in a recent overview of systematic reviews of HCP barriers and facilitators to DHIs, involving health workers in DHI design was identified as a common facilitator to utilisation of DHIs across all 108 primary systematic reviews ^29^. Beyond the usability of the digital platform, health workers identified challenges regarding its interoperability in the context of a health system where information and communications technology (ICT) is not standardised or harmonised. Not limited to our study, interoperability of DHIs is a global problem ^19, 34^, which requires implementation of national data interoperability standards in order to be addressed ^35^.

Similar to recent systematic review findings by Ferrel-Yui *et al.* ^15^, workforce capacity issues including HCP-patient ratios and time to deliver in the context of the return to in-person programmes acted as barriers to implementation. Frederix *et al.* ^36^, argue that DHIs need to be blended into current practices, rather than being an “add on” to existing delivery models. Ultimately, implementation of digital CR requires dedicated resources, including staff who are equipped with skills and competencies in digital health delivery ^12^. Our findings show that limited experience and training in digital health was an obstacle to implementation with similar barriers reported internationally ^19, 37^. These findings point to the need for greater integration of digital health competencies into the undergraduate and postgraduate curriculum as well as real-time, on the job training and support ^16, 38^.

Health workers reported uncertainties regarding patient suitability for virtual care; these related to low digital literacy levels, limited access to technology and broadband, and the patient’s CVD risk profile. These findings are not surprising given that digital CR studies tend to focus on homogenous populations, where the majority of participants are male, and younger (<63 years) ^11, 39^. To determine which patients may be best served by digital CRs, future research needs to be inclusive of oppressed and minoritized groups, e.g., people of colour, women, and the elderly. Furthermore, we believe these uncertainties could be addressed by conducting a rigorous assessment of patient needs, including their digital literacy and access to technology and broadband, in advance of DHI deployment. This will ensure that adequate supports are in place to enhance participation ^19^.

While there was widespread agreement that patients should be offered digital CR beyond the pandemic, referring health workers expressed safety concerns about delivering exercise remotely to high CVD risk patients. These safety concerns are mirrored internationally ^14, 40^, and could be addressed by greater use of remote monitoring technologies and sharing of good practice for the use of digital CR in high-risk patients ^5, 40^. Notwithstanding the importance of patient preference, health workers identified that hybrid CR, which combines in-person and remote components, could be a promising opportunity to broaden CR access ^41^. However, essential to this approach is effective integration of care between digital CR and in-person CR, and further research is required to understand how to optimally achieve this.

We observed similarities between our findings and the recent study by Kenny *et al*. ^42^ exploring patient experiences of MySláinte and a similar digital CR intervention. Specifically, health workers efforts to engage and support patients through developing interpersonal relationships, for example being more accessible and the provision of technical support, increased engagement. These findings suggest that DHIs do not necessarily compromise the patient-heath worker relationship, a commonly held perception among health workers ^43^. Furthermore, health workers identified that limited opportunities for peer support negatively affected patient engagement. A challenge, which health workers suggested, could potentially be overcome through a hybrid CR delivery model. Finally, health workers identified the importance of partner and family support in engaging patients to use the technology, a factor which is known to improve DHI use by patients ^15, 42^.

### Considerations for future implementation

Identifying strategies for effective implementation of digital health are a national and international priority ^19, 44, 45^. Based on our study findings, which included health workers views on factors to help inform future implementation, we developed a summary of key considerations to assist with future implementation efforts (see Table 3). Many of these considerations are consistent with solutions identified in the recent World Heart Foundation roadmap for digital health in cardiology ^19^, thus emphasising the relevance of this roadmap for digital CR. Considerations include actions at multiple levels from the digital intervention, to the patient, the health worker, and the broader health system. Importantly, as barriers and facilitators to implementation of digital CR are interdependent, these considerations should not be viewed in isolation but rather as part of a whole systems approach to implementation. Currently, findings from this study are informing the development and implementation of a self-management, mHealth intervention for the secondary prevention of CVD ^46^.

**Table 3.** Key considerations for the future implementation of digital cardiac rehabilitation.

Level	Considerations for future implementation of digital cardiac rehabilitation
Digital Health Intervention	Digital health interventions should: • Incorporate evidence-based core components and be delivered using a standardised approach • Be delivered by a skilled inter-professional team including nurse prescribers • Be designed with end user involvement (Patients and health workers) • Be integrated with in-person programmes and offered as part of a hybrid approach to care
Patient	Patients should: • Be offered a choice of CR delivery options, including digital programmes • Be provided with access (equipment and broadband) and the necessary skills and supports to use technology, which includes involving the family
Health Workers	Health workers should be: • Equipped with the necessary skills and competencies to deliver digital CR • Provided with adequate resources including staff, administrative support, time and technological support • Engaged early in the implementation process to ensure barriers to implementation are addressed • Provided with clear guidance on how to appropriately risk stratify and monitor patients remotely
Health System	At a systems level there needs to be: • Approval and support by the organisational leadership team including Cardiologists • Compliance with general data protection regulations and other relevant regulatory standards • Long-term funding to develop technology that minimises workload whilst also ensuring that there is an adequate workforce to deliver • A focus on patient centred care, ensuring seamless integration of care across all CR delivery modalities • A robust digital infrastructure, with greater interoperability • A focus on developing implementation strategies, which address context specific barriers.

### Limitations

This study has some potential limitations. Due to funding, this study was conducted 6-months after patients had completed MySláinte, which may have impacted HCP recollection of their experiences of implementation. However, it is also likely that having this time offered an opportunity for reflection on implementation efforts and achievement of programme goals, thus enhancing the richness of the data collected. We recognise that the context of COVID-19 may have influenced HCP perspectives of DHIs and therefore, factors influencing implementation may not be as relevant in the post pandemic era. Furthermore, as this was a nested qualitative study of a digital CR intervention in one region, we acknowledge that our findings may differ to other digital CR interventions. Nonetheless, we found similarities in our results to international data on DHIs in CVD care and CR ^13, 15, 19^. Understanding health workers perspectives of digital CR is a growing area of research and therefore future research inclusive of other health workers (for example physicians and psychologists) is required. This study was part of a multi-method evaluation of MySláinte, and while previous research has examined clinical outcomes ^20^ and patient experiences ^42^, there is also a need to consider cost effectiveness, an often neglected area in DHI research ^47^. Finally, while the aim of this study was to identify contextual factors influencing implementation of MySláinte, future research should align these factors to implementation outcomes (e.g., fidelity) to help determine which factors matter most.

## Conclusion

With an increasing emphasis being placed on offering patients a range of CR options, successful implementation of DHIs is contingent upon a comprehensive understanding of the challenges and opportunities faced by health workers. Leveraging on the practical experiences of implementing a digital CR intervention during the COVID-19 pandemic, we identified a number of factors which may help inform future strategies to enable scale up and integration into clinical practice. Findings from this study highlight how factors influencing implementation of digital CR are interconnected. Therefore, multi-level implementation strategies are required if the true potential of digital health in improving equitable CR access, participation and patient outcomes is to be realised.

## Ethics and consent

Ethical approval was obtained from the Galway Clinical Research Ethics Committee (Ref. C.A 2689) on the 27 ^th^ of August 2021. Informed written consent, was obtained by providing participants with a description of the research along with a participant information sheet.

## Data Availability

Study participants did not give consent for their data to be shared in a public repository. The information leaflet they received stated that their data would be anonymised and reported in aggregate. This was deemed necessary as the study is reporting on a qualitative study with a small number of participants, where they may be easily identified. Open science framework: Factors influencing implementation of digital cardiac rehabilitation: A qualitative analysis of health workers perspectives. DOI: https://doi.org/10.17605/OSF.IO/Z9MSX ^48^ This paper contains the following extended data: Supplementary file 1. Figure 1. Factors influencing implementation of Croí MySláinte across multiple levels Supplementary file 2. Interview topic guide Supplementary file 3. Summary of perceived barriers and facilitators to implementation of Croí MySláinte Data are available under the terms of the Creative Commons Attribution 4.0 International license (CC-BY 4.0)( https://creativecommons.org/licenses/by/4.0/). The study is reported in accordance with the Consolidated Criteria for Reporting Qualitative Research (COREQ) ^23^.
