# Supplementary material for: Factors influencing implementation of digital cardiac rehabilitation: A qualitative analysis of health workers perspectives
Source: HRB Open Res. 2024 Jul 26;7:50. [Version 1] doi: 10.12688/hrbopenres.13929.1 (PMC11464961; doi:10.12688/hrbopenres.13929.1)
Supplement: Peer review history

# Reviewer report: Reviewer response for version 1

**DOI:** 10.21956/hrbopenres.15283.r42523

**Published:** 2024-10-09

**Authors:** Ahmad M Osailan

**Affiliations:** 1 Prince Sattam Bin Abdulaziz University, University in Al-Kharj, Saudi Arabia

The article "Factors influencing implementation of digital cardiac rehabilitation: A qualitative analysis of health workers perspectives" offers valuable insights into the barriers and facilitators to implementing digital cardiac rehabilitation (CR) programs like MySláinte. While the study provides a strong basis for understanding the factors at play, there are a few areas where improvements could enhance the clarity and impact of the paper.

**Introduction Clarity**: The introduction gives a broad view of digital CR and the barriers to its adoption, but it would benefit from a more concise explanation of the MySláinte program. It lacks sufficient background on how the program operates, which is vital for readers unfamiliar with it. A more detailed explanation of its key features—such as how it integrates technology with healthcare—would better ground the reader for the analysis to follow.

**Separation of Barriers and Facilitators**: The results section discusses barriers and facilitators across multiple domains, sometimes muddling the analysis. Separating these into distinct sections or using tables to list the pure barriers and facilitators would make the discussion more digestible. Additionally, providing direct illustrative quotes alongside the barriers and facilitators in the table would allow for more engaging content and help emphasize the real-world impact, making the audience feel more connected and involved in the discussion.

**Methodology** The methodology is robust, but more detail on the specific roles and experiences of participants could enhance the credibility of the findings. For example, although it is mentioned that health workers from various roles were interviewed, discussing more about how their different perspectives (e.g., frontline workers vs. managerial staff) influenced their views on digital CR would make the analysis richer.

**Deeper Analysis of Specific Challenges**: While the paper touches upon critical challenges like digital infrastructure, workload, and training gaps, it could benefit from a more analysis of how these barriers were overcome or exacerbated during the MySláinte implementation. For example, more detailed examples of how specific barriers, like the lack of interoperability between systems, played out in practice would help connect the findings more directly to actionable solutions.

**Discussion:** The discussion mixes barriers and facilitators in a way that could be more organized. Grouping the facilitators and obstacles into distinct paragraphs or sections could improve readability. Additionally, providing more information on the technological challenges faced by health workers, specifically the functionality of the Moodle platform used for the program, could give a clearer view of the specific technical limitations that impacted implementation.

**Recommendations and Conclusion**: The recommendations for future implementations are solid but could be strengthened by offering more specific, practical solutions to some identified problems. For instance, the need for "a robust digital infrastructure, with greater interoperability" is mentioned but could be expanded with examples of successful implementations from other contexts. In summary, the paper offers substantial contributions to understanding digital CR but could improve its impact through clearer structure and deeper engagement with its identified challenges. Adding more detail about the MySláinte program, separating barriers and facilitators, and providing practical recommendations would make the paper more effective and accessible.

Is the work clearly and accurately presented and does it cite the current literature?

Partly

If applicable, is the statistical analysis and its interpretation appropriate?

Not applicable

Are all the source data underlying the results available to ensure full reproducibility?

Yes

Is the study design appropriate and is the work technically sound?

Yes

Are the conclusions drawn adequately supported by the results?

Yes

Are sufficient details of methods and analysis provided to allow replication by others?

Yes

Reviewer Expertise:

Cardiac Rehabilitation

I confirm that I have read this submission and believe that I have an appropriate level of expertise to confirm that it is of an acceptable scientific standard, however I have significant reservations, as outlined above.

# Reviewer report: Reviewer response for version 1

**DOI:** 10.21956/hrbopenres.15283.r41880

**Published:** 2024-09-27

**Authors:** LaPrincess Brewer, Grace Patrice Anyetei-Anum

**Affiliations:** 1 Mayo Clinic College of Medicine, Rochester, MN, USA

The manuscript titled, “ ***Factors influencing implementation of digital cardiac***

***Rehabilitation: A qualitative analysis of health workers p*** ***erspectives***” is an interesting manuscript examining the perspectives of health workers on factors influencing the implementation of a cardiac rehabilitation (CR) program. These factors include: i) characteristics of health workers, ii) features of the CR program, iii) the relationship of the external and internal environment on the delivery of the CR program and iv) the implementation process of the CR program. The authors’ key finding was that factors affecting the implementation of digital CR are multi-layered; with a corresponding conclusion that to maximize beneficial use of digital CR, a well-rounded, multi-step approach is essential.

 The strengths of the manuscript are in its use of the updated Consolidated Framework for Implementation Research (CFIR), a sound implementation science framework to direct qualitative data collection and analysis. Another strength of the paper is that it contributes to a growing body of knowledge on health workers perspectives on the use of digital CR – an area currently lacking in the literature. Overall, the paper has valuable insights that can be utilized to inform the implementation of digital CR in Europe and worldwide, potentially revolutionizing the field. This research also has the potential to significantly increase access to CR, particularly for underrepresented groups. Main concerns lie in the Introduction, Results and Discussion.

 MAJOR REVISIONS:

 Introduction

1. Although the authors cite previous work on the MySláinte interface in the introduction, it would be helpful for readers to gain more insight directly from this specific paper. As such, the authors should elaborate further on how the MySláinte app works to give readers a deeper understanding of their digital CR platform in the Methods section.

Results

1. The authors should separate the barriers and facilitators of the domains (innovation, outer setting, inner setting, individuals, implementation process) into tables with rows showcasing the pure barriers, pure facilitators or both, each with an illustrative quote.

Discussion

1. The authors should provide more details on how the existing platform Moodle works and how it is similar and different from MySláinte.

1. Authors should discuss barriers to the implementation of MySláinte in one paragraph and facilitators in another paragraph to enhance readability.
2. For Figure 1, the authors should provide more information on the factors listed in both the inner and outer setting directly. For example, how does policy and law directly/indirectly impact the implementation of digital CR?
3. In Table 3, the statement “A robust digital infrastructure, with greater interoperability” is lofty and broad and needs more details.

MINOR REVISIONS

1. In Figure 1, the authors should further describe the meaning of (+) and (-). For example, facilitators only should be designated (+), barriers only (-) and both facilitators and barriers indicated by (+/-).
2. Authors should include the dates of the original trial, recruitment and data collection of the current study.
3. Authors should include reference (Adedinsewo et al. 2023[Ref -1]) for the sentence “…future research needs to be inclusive of oppressed and minoritized groups, e.g., people of colour, women, and the elderly.” 
4. Consider the following publications for references for digital CR and its implementation.
  1. Adedinsewo D, et al. (2023 [Ref-1]) Health Disparities, Clinical Trials, and the Digital Divide. Mayo Clin Proc. 2023 Dec. PMID: 38044003; PMCID: PMC10825871.
  2. Ravn MB, et al. Understanding Facilitators and Challenges to Care Transition in Cardiac Rehabilitation: Perspectives and Assumptions of Healthcare Professionals. Glob Qual Nurs Res. (2023 [Ref-2]) Dec. PMID: 38107551; PMCID: PMC10722928.
  3. Kim C, et al. A survey of the perceptions of barriers to and facilitators of cardiac rehabilitation in healthcare providers and policy stakeholders. BMC Health Serv Res. 2022 Aug. PMID: 35932056; PMCID: PMC9356496.
5. Given its recent release, recommend citing the more recent scientific statement on core components of CR:

1. Brown TM, et al. 2024 (Ref - 4) Sep 24. Epub ahead of print. PMID: 39315436.

Is the work clearly and accurately presented and does it cite the current literature?

Yes

If applicable, is the statistical analysis and its interpretation appropriate?

Yes

Are all the source data underlying the results available to ensure full reproducibility?

Yes

Is the study design appropriate and is the work technically sound?

Yes

Are the conclusions drawn adequately supported by the results?

Yes

Are sufficient details of methods and analysis provided to allow replication by others?

Yes

Reviewer Expertise:

Cardiac rehabilitation, health equity, preventive cardiology

We confirm that we have read this submission and believe that we have an appropriate level of expertise to confirm that it is of an acceptable scientific standard, however we have significant reservations, as outlined above.

## References

[1] : Health Disparities, Clinical Trials, and the Digital Divide. *Mayo Clin Proc* .2023;98(12) : 10.1016/j.mayocp.2023.05.003 1875-1887 10.1016/j.mayocp.2023.05.003 38044003 PMC10825871

[2] : Understanding Facilitators and Challenges to Care Transition in Cardiac Rehabilitation: Perspectives and Assumptions of Healthcare Professionals. *Glob Qual Nurs Res* .2023;10: 10.1177/23333936231217844 23333936231217844 10.1177/23333936231217844 38107551 PMC10722928

[3] : A survey of the perceptions of barriers to and facilitators of cardiac rehabilitation in healthcare providers and policy stakeholders. *BMC Health Serv Res* .2022;22(1) : 10.1186/s12913-022-08298-3 999 10.1186/s12913-022-08298-3 35932056 PMC9356496

[4] : Core Components of Cardiac Rehabilitation Programs: 2024 Update: A Scientific Statement From the American Heart Association and the American Association of Cardiovascular and Pulmonary Rehabilitation. *Circulation* .2024; 10.1161/CIR.0000000000001289 10.1161/CIR.0000000000001289 39315436

# Reviewer report: Reviewer response for version 1

**DOI:** 10.21956/hrbopenres.15283.r41672

**Published:** 2024-08-21

**Authors:** Nilay Shah

**Affiliations:** 1 Northwestern University Feinberg School of Medicine, Chicago, IL, USA

In this manuscript, the authors present the findings of a qualitative analysis of the implementation of MySlainte, a digital cardiac rehabilitation program launched during the COVID-19 pandemic. The implementation evaluation follows the CFIR framework and focuses on health care professional perspectives of facilitators and barriers to implementation of the digital CR program. This analysis addresses an important and timely topic and the methodological approach is sound and grounded in a well-established framework in implementation science. The manuscript is clear and very well-written. Several comments are offered:

  

1. In the Introduction, it would be helpful to briefly detail the medical conditions for which cardiac rehabilitation is recommended.
2. The interpretation of the results would be facilitated by some greater detail of the health care professional roles. Though the HCP titles are provided, to understand how each interviewee interacts with the digital CR program, it would be important to understand how each person in the named roles specifically interacts with the digital CR program (for example, referring patients to CR, delivering the CR program, administering or managing the CR program, etc.)
3. In the Results – Innovation section, the authors refer to “poor functionality of the online platform.” Can the authors expand on this description, specifically regarding what aspects of the platform functioned poorly?
4. In the Results – Inner Setting section, the authors provide example quotes regarding “access to available resources… [that] facilitated implementation.” Did any of the interviewees provide additional detail about the work needed to implement the MySlainte program? This would materially aid the audience to understand the specific aspects or steps of implementation they might need to follow to adapt this CR approach
5. Although the authors do briefly address the context of the COVID-19 pandemic in the Discussion, this seems to be a considerable factor influencing the implementation of the digital CR program. I think their Discussion narrative would benefit from adding their interpretation and contextualization, specifically regarding what might people have to do to adapt the digital CR program outside of the COVID-19 pandemic context?
  This comment is offered because of some of the participants’ responses alluded to an environment in which the deployment of the digital CR program was actually facilitated and accelerated by virtue of the simple necessity of doing so given the pandemic-related limitations for in-person CR (for example, in the Outer Setting section, one participants stated “I think the fact that COVID happened we were pushed to do things… quicker”).
  Could the authors synthesize what aspects of the MySlainte intervention were specifically facilitators to the program given the COVID-19 pandemic outer setting? And, where such facilitators don’t exist (e.g., now in-person CR is allowed again), what might a digital CR program implementation staff have to do differently?

Is the work clearly and accurately presented and does it cite the current literature?

Yes

If applicable, is the statistical analysis and its interpretation appropriate?

Not applicable

Are all the source data underlying the results available to ensure full reproducibility?

Yes

Is the study design appropriate and is the work technically sound?

Yes

Are the conclusions drawn adequately supported by the results?

Yes

Are sufficient details of methods and analysis provided to allow replication by others?

Yes

Reviewer Expertise:

Cardiovascular disease prevention

I confirm that I have read this submission and believe that I have an appropriate level of expertise to confirm that it is of an acceptable scientific standard.
